# Aminochrome as preclinical model for Parkinson's disease

**DOI:** 10.18632/oncotarget.18353

**Published:** 2017-06-02

**Authors:** Juan Segura-Aguilar

**Affiliations:** Molecular and Clinical Pharmacology, ICBM, Faculty of Medicine, University of Chile, Santiago, Chile

**Keywords:** neurodegeneration, dopamine, neuromelanin, DT-diaphorase, glutathione transferase M2-2

It is essential the existence of suitable preclinical models to elucidate the molecular mechanism of a disease and to test potential drugs and therapies. One of the major problems to discover new pharmacological therapies in Parkinson's disease is the lack of suitable preclinical model. This explains why L-dopa is still the most effective drug despite the severe side effects observed after 4-6 years of treatment. There is a long list of clinical studies failed despite being based on robust preclinical results obtained with exogenous neurotoxins as preclinical model. The use of preclinical models based on exogenous neurotoxins (6-hydroxydopamine, 1-methyl-4-phenyl-1,2,3,6-tetrahydropyridine (MPTP) or rotenone) is an important problem to translate successful results to clinical studies and new therapies in Parkinson's disease [1]. The discovery that glial cell-derived neurotrophic factor (GDNF) preserved nigrostriatal circuitry and reversed motor disability in animals lesioned with systemic MPTP or unilateral striatal injections of 6-hydroxydopamine opened a huge hope to obtain a new treatment in Parkinson's disease. It was expected that GDNF halts the progression of the disease and regenerate dopaminergic neurons. However, clinical studies with GDNF revealed no significant difference between control and patients [2]. The question is why we cannot translate successful preclinical studies performed with exogenous neurotoxins to clinical studies. In my opinion, the failure to translate results from preclinical to clinical studies depends on (i) the use of preclinical models based on exogenous neurotoxins that do not replicate what happen in Parkinson's disease. These preclinical models induce an extremely rapid and massive degeneration of the nigrostriatal dopaminergic neurons. For example MPTP induces a severe Parkinsonism in just 3 days in humans exposed to this neurotoxin while the degenerative process in Parkinson's disease takes years before the motor symptoms are evident. In addition, the progression of the disease is also extremely slow in comparison to the rapid effects of these exogenous neurotoxins [3]; and (ii) the mechanism that triggers the degeneration of dopaminergic neurons containing neuromelanin in Parkinson's disease is still unknown. However, there is a general agreement in the scientific community that mitochondrial dysfunction, generation of neurotoxic oligomers of alpha-synuclein, protein degradation dysfunction of both proteasomal and lysosomal systems, oxidative stress, neuroinflammation and endoplasmic reticulum stress are direct involved in the degenerative process of the nigrostriatal system. But the problem is what triggers these mechanisms in dopaminergic neurons containing neuromelanin in the nigrostriatal neurons [3].

Recently, we published that aminochrome can be used as a new preclinical model for Parkinson's disease. The unilateral injection of aminochrome in the striatum induces contralateral behavior without significant loss of dopaminergic neurons accompanied with a significant decrease in dopamine release and a significant increase in GABA levels, generating an imbalance of neurotransmitters in the basal ganglia. Aminochrome induces mitochondrial dysfunction resulting in a significant decrease in ATP level, explaining the significant decrease in the amount of synaptic vesicles in the terminals and a decrease in dopamine release since both axonal transport of vesicles for neurotransmission and dopamine release require ATP [4].

Aminochrome is an o-quinone formed during dopamine oxidation to neuromelanin in dopaminergic neurons containing neuromelanin. It has been demonstrated that aminochrome induces mitochondrial dysfunction, generation of neurotoxic oligomers of alpha-synuclein, protein degradation dysfunction of both proteasomal and lysosomal systems, oxidative and endoplasmic reticulum stress (reactions 3-8, Figure 1) [3]. Dopamine oxidation to neuromelanin involves the formation of several o-quinones (dopamine → dopamine o-quinone → aminochrome → 5,6-indolequinone → neuromelanin; reactions 1, 2, 12, 12, 14, Figure 1). This is a normal pathway since healthy senior individuals have intact their dopaminergic neurons containing neuromelanin [3]. Aminochrome participates in neurotoxic reactions by forming adducts with proteins and/or be one-electron reduced by flavoenzymes. However, there are two enzymes which prevent aminochrome neurotoxicity: (i) DT-diaphorase, expressed both in dopaminergic neurons and astrocytes, catalyzes the two electron reduction of aminochrome to leukoaminochrome (reaction 9; Figure 1) preventing aminochrome induced cell death, mitochondrial dysfunction; protein degradation dysfunction both proteasomal and lysosomal, oxidative stress, and formation of neurotoxic alpha-synuclein oligomers [3]; and (ii) human glutathione transferase M2-2 (GSTM2), expressed only in astrocytes, catalyzes GSH conjugation of both aminochrome and its precursor dopamine o-quinone (reactions 10 and 11, Figure 1) to non-reactive products [for review see reference 5]. Degradation product of the conjugate of dopamine o-quinone has been found in human cerebrospinal fluid and neuromelanin [5]. GSTM2 prevents aminochrome-induced cell death, autophagy and lysosome dysfunction in astrocytes [6]. Interestingly, astrocytes seem to protect dopaminergic neurons against aminochrome neurotoxicity by secreting GSTM2 into the extracellular space. Interestingly, dopaminergic neurons are able to internalize GSTM2 into the cytosol in order to protect dopaminergic neurons against aminochrome neurotoxicity [5, 7].

**Figure 1:**
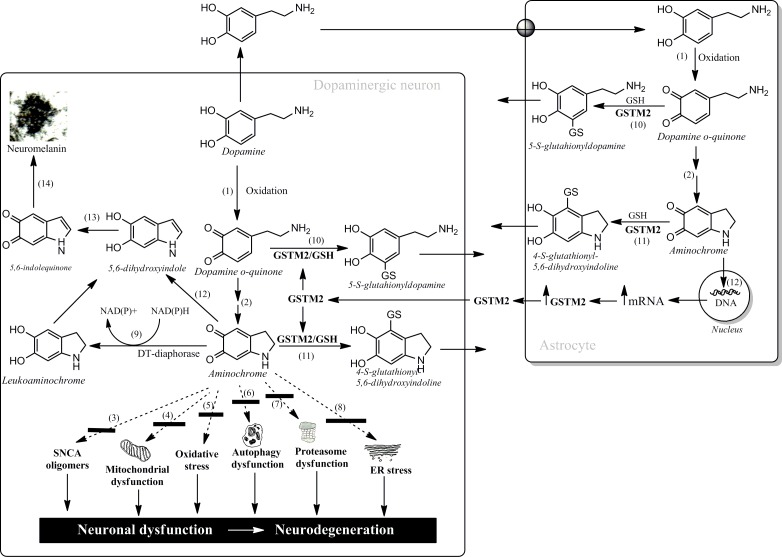
Neurotoxic and neuroprotective reactions of aminochrome

In conclusion, we need a new preclinical model that replicate what happen in the degenerative process in Parkinson's disease to discover new drugs, which not only alleviate L-dopa side effects such dyskinesia but halt the progression of the disease. The advantages of aminochrome as preclinical model for Parkinson's disease are (i) aminochrome is formed inside of dopaminergic neurons containing neuromelanin lost during the disease; (ii) aminochrome triggers mitochondria dysfunction, formation of alpha-synuclein neurotoxic oligomers, protein degradation dysfunction, oxidative stress and endoplasmic reticulum stress.

## References

[1] Athauda D, Foltynie T (2014). Nat Rev Neurol.

[2] Olanow CW (2015). Mov Disord.

[3] Segura-Aguilar J (2014). J Neurochem.

[4] Herrera A (2016). Cell Mol Life Sci.

[5] Segura-Aguilar J (2015). Neural Regen Res.

[6] Huenchuguala S (2014). Autophagy.

[7] Cuevas C (2015). Neurotox Res.

